# Unlocking the Potential of Circulating miRNAs in the Breast Cancer Neoadjuvant Setting: A Systematic Review and Meta-Analysis

**DOI:** 10.3390/cancers15133424

**Published:** 2023-06-30

**Authors:** Paola Tiberio, Mariangela Gaudio, Silvia Belloni, Sebastiano Pindilli, Chiara Benvenuti, Flavia Jacobs, Giuseppe Saltalamacchia, Alberto Zambelli, Armando Santoro, Rita De Sanctis

**Affiliations:** 1 Medical Oncology and Hematology Unit, IRCCS Humanitas Research Hospital, 20089 Rozzano, Italy; paola.tiberio@cancercenter.humanitas.it (P.T.); mariangela.gaudio@cancercenter.humanitas.it (M.G.); chiara.benvenuti@cancercenter.humanitas.it (C.B.); flavia.jacobs@cancercenter.humanitas.it (F.J.); giuseppe.saltalamacchia@cancercenter.humanitas.it (G.S.); alberto.zambelli@hunimed.eu (A.Z.); armando.santoro@cancercenter.humanitas.it (A.S.); 2 Department of Biomedical Sciences, Humanitas University, 20072 Pieve Emanuele, Italy; sebastiano.pindilli@st.hunimed.eu; 3 Educational and Research Unit, IRCCS Humanitas Research Hospital, 20089 Rozzano, Italy

**Keywords:** microRNAs, circulating miRNAs, breast cancer, neoadjuvant chemotherapy, pathological complete response, miR-21-5p, miR-155-5p

## Abstract

**Simple Summary:**

In recent decades, neoadjuvant chemotherapy has proven to be a viable therapeutic option, particularly in cases of high-risk early or locally advanced breast cancer, for reducing tumor size, improving surgical outcomes, and evaluating full histological responses. Thus, individualizing post-neoadjuvant therapy has the potential to enhance prognosis. However, individual responses to therapy and long-term prognosis remain highly unpredictable. The current scenario requires the identification of biomarkers that accurately forecast responses to neoadjuvant therapy and identify patients who will not benefit from standard regimens. Circulating microRNAs have emerged as potential non-invasive biomarkers for breast cancer management. However, discrepancies between different studies currently hamper the implementation of circulating microRNAs, as a significant biomarker, in clinical practice.

**Abstract:**

The potential role of circulating microRNAs (miRNAs) as biomarkers in breast cancer (BC) management has been widely reported. However, the numerous discrepancies between studies in this regard hinders the implementation of circulating miRNAs in routine clinical practice. In the context of BC patients undergoing neoadjuvant chemotherapy (NAC), the possibility of predicting NAC response may lead to prognostic improvements by individualizing post-neoadjuvant therapy. In this context, the present meta-analysis aims to clarify circulating miRNAs’ predictive role with respect to NAC response among BC patients. We conducted a comprehensive literature search on five medical databases until 16 February 2023. We pooled the effect sizes of each study by applying a random-effects model. Cochran’s Q test (*p*-level of significance set at 0.05) scores and I^2^ values were assessed to determine between-study heterogeneity. The PROBAST (Prediction Model Risk of Bias Assessment Tool) tool was used to evaluate the selected studies’ risk of bias. Overall, our findings support the hypothesis that circulating miRNAs, specifically miR-21-5p and miR-155-5p, may act as predictive biomarkers in the neoadjuvant setting among BC patients. However, due to the limited number of studies included in this meta-analysis and the high degrees of clinical and statistical heterogeneity, further research is required to confirm the predictive power of circulating miR-21-5p and miR-155-5p.

## 1. Introduction

Breast cancer (BC) is a heterogeneous disease with distinct molecular subtypes characterized by different prognoses and sensitivity to specific treatments [1]. The presence or absence of specific hormone receptors (HRs), such as oestrogen and progesterone receptors (ERs and PgRs, respectively), and the overexpression of the human epidermal growth factor receptor 2 (HER2) are the factors that determine the simplified immunohistochemistry-based BC molecular classifications [2]. Understanding each BC subtype is crucial for the development of individualized treatment strategies. Additionally, the use of neoadjuvant chemotherapy (NAC) has expanded in recent decades, especially with respect to triple-negative (TN) and HER2-positive BC, which exhibit aggressive clinical-pathological features and poor prognosis. Preoperative treatment is required not only to reduce tumor size and optimize surgical outcomes but also to evaluate the pathological complete response (pCR), which is defined as ypT0/is ypN0 [3]. Indeed, pivotal studies have demonstrated the long-term prognostic significance of pCR per se both in terms of invasive disease-free survival and overall survival [4,5]. Moreover, defining pathological responses could aid in identifying patients with a poorer prognosis, allowing for personalized post-neoadjuvant therapy and potentially improving prognosis. However, individual responses to NAC and long-term prognosis remain highly unpredictable. Therefore, there is an urgent need for the identification of biomarkers that can predict responses to NAC and identify patients who will not benefit from standard regimens. This approach can avoid unnecessary toxicities and pave the way for the escalation or de-escalation of personalized treatment.

MicroRNAs (miRNAs) are short, highly conserved, non-coding RNA molecules that play crucial roles as gene regulatory networks, in which they mediate post-transcriptional gene silencing. Based on their regulation and the functions of their target genes, miRNAs may operate as oncosuppressors or oncogenes [6]. MiRNA biogenesis is a multi-enzyme process involving the initial generation of a primary miRNA (pri-miRNA), which is then capped, spliced, polyadenylated, and cleaved into a precursor miRNA (pre-miRNA) [7]. Consequently, from the 5′ and 3′ ends of the pre-miRNA, an miRNA duplex composed of two strands (i.e., 5p and 3p) is generated [8]. The guide strand is subsequently loaded into the miRNA-induced silencing complex (miRISC), which targets specific messenger RNAs (mRNAs) for degradation or translational repression, while the passenger strand can be degraded or incorporated into the miRISC complex based on tissue or cell type [9]. The miRNA-loaded RISC then scans the messenger RNA (mRNA) molecules to identify complementary sequences, leading to the degradation of the targeted mRNA, the inhibition of its translation into protein, or an increase in the translation of target mRNA [8,10]. This mechanism is crucial in regulating post-transcriptional gene expression and is involved in various biological processes, such as cell development, differentiation, and function and the pathogenesis of various human diseases, including cancer [6,8,11,12].

Moreover, miRNAs have also been identified as potential diagnostic and prognostic biomarkers for cancer, as their expression profiles are often altered in tumor tissues compared to normal ones [6,13,14]. The discovery of miRNAs in extracellular fluids (including blood, serum, plasma, urine, saliva, seminal fluid, and pleural effusion) as well has opened up new possibilities for their use as non-invasive biomarkers for cancer diagnosis and prognosis [15]. In fact, miRNAs can be released in the circulation as a passive consequence of cell death or due to active secretion [8,16,17,18,19,20] in a stable form protected from endogenous RNAses (associated with proteins or contained in exosomes, microvesicles, or apoptotic bodies), thus highlighting the potential of circulating miRNA as non-invasive biomarkers for different tumor types, including BC [21,22,23,24].

In a recent review, we discussed the diagnostic, predictive, and prognostic significance of different circulating miRNAs, such as oncogene-like miR-21-5p and tumor suppressor-like miR-34a-5p and miR-let-7a-5p, as potential non-invasive biomarkers for BC patients undergoing NAC [25]. As reported in the literature, circulating miR-21-5p is the most extensively investigated miRNA with respect to BC, and it has been suggested to be a promising predictive biomarker for NAC response [26,27,28]. However, a high level of discrepancy throughout different studies was reported in our review [25], thus limiting the incorporation of circulating miRNAs as biomarkers into routine clinical practice. In general, predictive factors have numerous potential applications, including assisting in treatment and lifestyle decisions, improving individual risk prediction, identifying novel targets for new treatments, and improving the design and analysis of randomized trials [29,30]. In this context, a meta-analysis is required to provide an overall quantitative synthesis of the available evidence in order to clarify the predictive role of circulating miRNAs with regard to NAC response among BC patients.

## 2. Materials and Methods

Our study was conducted based on the following review question: “Which circulating miRNAs are currently available in predicting the response to NAC in adult women with BC?”. We conducted a systematic review and meta-analysis in compliance with the Preferred Reporting Items for Systematic Reviews and Meta-Analyses (PRISMA) statement and flow chart [31]. For our study, we applied the Cochrane-recommended methodology for conducting systematic reviews and meta-analyses of prognostic factor studies [32]. Although the present systematic review has not been recorded in the Prospero database, we conducted a comprehensive search on Prospero before initiating our analyses. Specifically, we searched for existing systematic reviews with similar parameters (i.e., the use of circulating miRNAs to predict NAC response among BC patients) without finding any ongoing or published meta-analyses.

### 2.1. Article Selection and Eligibility Criteria

In order to identify relevant studies, we conducted a comprehensive literature search, without time limits, on PubMed, Cochrane Library, EMBASE, Scopus, and Web of Science for all relevant studies published until 16 February 2023. We also retrieved abstracts from major international conferences from the last two years (namely, American Society of Clinical Oncology (ASCO), European Society of Medical Oncology (ESMO) and ESMO Breast, and San Antonio Breast Cancer Symposium (SABCS)) in order to identify potentially eligible unpublished studies. The article search was performed using the following search strategy: (Circulating OR Plasma OR Blood OR Serum) AND (microRNA OR miRNA) AND (breast AND Cancer) AND (neoadjuvant OR preoperative AND chemotherapy). In addition, other relevant studies were identified by manually searching for references of eligible publications. Additionally, a manual search was conducted on Google Scholar and ClinicalTrials.gov [33] as a complementary search. No language restrictions were applied in the database search so as to identify all relevant publications.

The review question and the studies’ selection were based on the Population, Index, Comparison, Outcome, Timing, and Setting (PICOTS) framework [34,35]: (a) population—women (age > 18 years) with BC undergoing any type of NAC; (b) index—prognostic factors, namely, circulating miRNAs levels; (c) comparator—not applicable; (d) outcome—pCR achievement; (e) timing—circulating miRNA levels evaluated before NAC or after the first two cycles of NAC; and (f) setting—medical oncology unit. For studies presenting scores or predictive models, the accuracy is reported as the area under the curve (AUC) or the odds ratios (ORs), and corresponding 95% confidence intervals (CIs) were considered. We included cohort studies and correlative analyses of randomized controlled trials (RCTs). Non-original articles (e.g., case reports, reviews, letters, and meta-analyses) were excluded. Articles published in languages other than English were thus omitted throughout the title and abstract selection process, regardless of whether they were significant with respect to the review’s aims, to reduce any potential linguistic misinterpretation.

### 2.2. Data Selection and Extraction

The literature search was undertaken independently by two authors by first reading the titles and abstracts of the identified papers. To guarantee the process’s reliability and consistency, the two authors discussed reasons for inclusion or exclusion and disagreements. The studies determined eligible after abstract screening were then independently assessed in full to accurately analyze the studies’ contents. The data gathered were recorded in a piloted, customized ‘Data Extraction Form’ created in Microsoft Excel for data synthesis evaluation [36]. Disagreements between the two authors throughout the data selection and extraction phase were handled through a consensus discussion with a third author. The CHARMS checklist (the Checklist for critical Appraisal and data extraction for systematic Reviews of prediction Modelling Studies) guided the study selection and data extraction processes [37] (Supplementary Figure S1).

### 2.3. Quality Appraisal

Two authors independently performed the quality appraisal of the studies. The PROBAST (Prediction model Risk of Bias Assessment Tool) tool was utilized to assess the risk of bias (ROB) and the applicability of predictive model research [38,39]. This assessment template was used to analyze the full-text studies that were determined eligible for our systematic review. PROBAST consists of 4 domains: (1) Participants, (2) Predictors, (3) Outcome, and (4) Analysis. The domains include twenty indicating questions, whose possible answers are as follows: yes (Y), probably yes (PY), no (N), probably no (PN), or no information (NI). The answer “yes” indicates low ROB, while the answer “no” indicates high ROB. PY and PN answers are provided to enable judgments when there is an insufficient amount of information with which to answer Y or N confidently [39]. Any disagreements between the two authors during the quality assessment process were resolved through discussion.

### 2.4. Statistical Analysis

We performed a meta-analysis to assess circulating miRNAs’ ability to predict response to NAC among BC patients, as more than five external validation studies were available for the same index prognostic model [40]. We quantitatively pooled the effect sizes of each study by applying the method of restricted maximum likelihood (REML) [41]. We calculated an average value of a prediction model’s performance using the effect sizes, CIs, and the standard error retrieved from the primary studies [42,43]. Given the substantial clinical heterogeneity of the studies, we used a random-effects model to account for within- and between-study variations [44]. To measure between-study heterogeneity, Cochran’s Q test (*p*-level of significance set at 0.05) and I^2^ indexes were applied. The following I^2^ cut-offs were considered to evaluate heterogeneity: 0–40% (an unimportant degree of heterogeneity), 30–60% (moderate), and higher than 50% (substantial and considerable heterogeneity) [31]. Based on the meta-analysis’s results, we performed subgroup analysis (categorical variables) or a meta-regression (continuous variables) to explore within- and between-study heterogeneity. STATA 16 software (StataCorp. 2019. Stata Statistical Software: Release 16. College Station, TX, USA: StataCorp LLC) was used for data analysis.

### 2.5. Measurements

The primary studies included in this systematic review employed diverse statistical methods, such as logistic regression analysis and receiver operating characteristic (ROC) curves, to assess the associations between circulating miRNA levels and pCR. These methods were utilized to estimate the predictive value and performance of miRNAs. Logistic regression analysis of the primary studies produced results in terms of ORs, whereas ROC curve analysis conveyed results as AUC measures. While the OR from logistic regression describes a biomarker’s association with clinical events, the AUC of the ROC curve (C-statistic) is commonly used to evaluate a biomarker’s discriminatory ability to predict an event [45,46]. Thus, ordinal logistic regression was considered to test the predictive value of miRNAs in response to NAC among BC patients; the AUC of the ROC curve was considered to determine the predictive accuracy of miRNAs in predicting NAC responses among BC patients. Since our analysis aimed to analyze the potential value of several biomarkers on different scales as continuous value covariates, we reported associations as OR per unit increase in the marker that describes the increased odds of an event per 1 standard deviation (SD) [45]. The OR was used to describe the expected change in odds for a unit increase in the predictor or the continuous effect of a predictor on the likelihood of one outcome occurring [47,48,49]. Thus, when the OR is less than one, increasing variable values correspond to decreasing event occurrence odds; when the OR exceeds one, improving variable values are correlated with increasing odds of the event occurring [50]. AUC is a valuable metric for assessing a test’s total diagnostic accuracy: an AUC of 0.5 indicates no discrimination for the predictive biomarker’s performance, a score of 0.7 to 0.8 is considered acceptable, a score of 0.8 to 0.9 is excellent, and a score greater than 0.9 is outstanding [51]. Considering the specificity of each effect size, whose applicability depends on the research question and study’s design, we performed separate meta-analyses by maintaining the original effect size measure expressed in the primary studies and preserving the two-dimensional nature of the underlying data [52]. The prognostic value of miR-21-5p and miR-155-5p was also investigated by converting the OR estimates into AUC values, assuming a similar study design [53]. Two prediction models resulted from our analysis (an OR regression model and model concerning the AUCs of the ROC curve).

## 3. Results

### 3.1. Characteristics of the Included Studies

**Figure 1:**
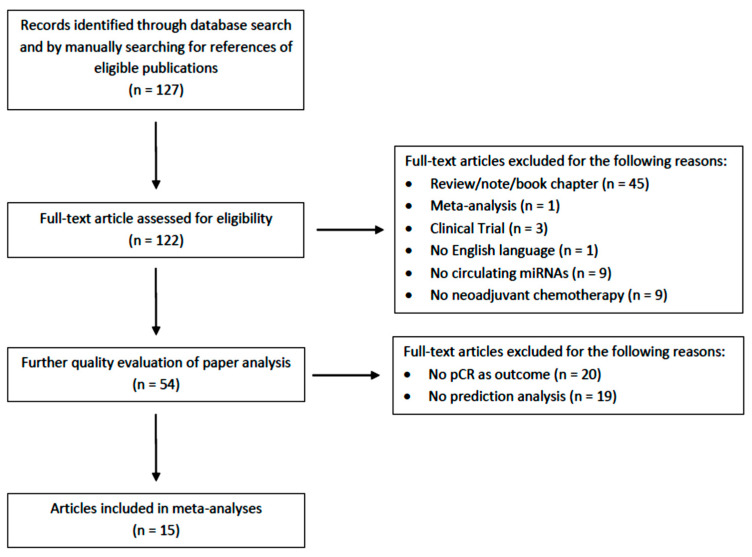
The literature search process, including the reasons for the articles’ exclusion.

A total of 127 records were identified from among the selected databases and conference proceedings by using the above-mentioned research criteria. After duplicate removal and the exclusion of non-relevant records, 15 studies were included in the present meta-analysis (Figure 1).

The main characteristics of the studies included in the meta-analyses are reported in Table 1. All the articles included were published between 2014 and 2022. Five studies were conducted using a mixed BC population and one was conducted using HER2-positive and TNBC patients, whereas the remaining investigations focused on a specific BC subtype (i.e., HER2-positive, HER2-negative, HR-positive, and Luminal B). The number of BC patients enrolled in each study varied from 32 to 435. BC patients underwent different neoadjuvant regimens based on the specific BC subtypes (mainly anthracycline- and taxane-based but also antiHER2 agents in the case of HER2-positive disease). All the analyses were performed using plasma or serum samples, except for the study by McGuire [54] and colleagues, which employed whole blood. Quantitative real-time polymerase chain reaction (qRT-PCR) was mostly used for comparing circulating miRNA levels between responders and not responders.

### 3.2. Risk of Bias of the Selected Studies

**Figure 2:**
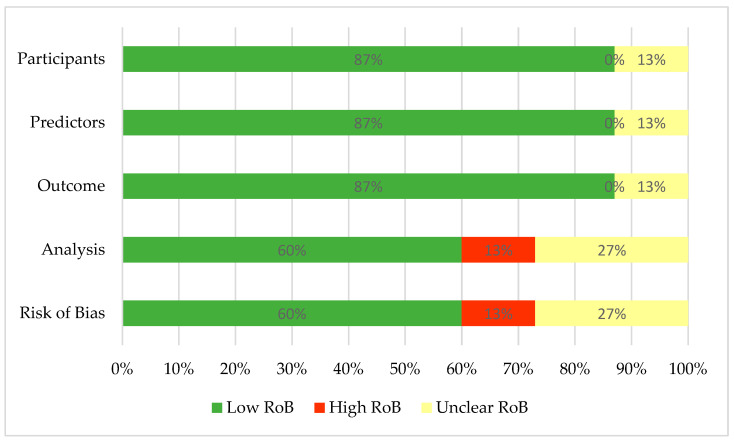
Summary of risk of bias assessment. RoB—risk of bias.

Overall, nine studies (60%) [26,28,55,56,58,59,60,62,63] received a “low ROB”, four studies (27%) [57,61,65,66] were assigned an “unclear ROB” rating, and two studies (13%) [54,64] were assigned a “high ROB”. The omission of appropriate statistical analysis for predicting model validation and information in the first three domains (i.e., participants, predictors, and outcome) was a frequent source of bias in terms of high and unclear judgment. Supplementary Table S1 and Figure 2 display the results of the risk of bias analysis.

### 3.3. Pooled Estimates of the Logistic Regression Models

**Figure 3:**
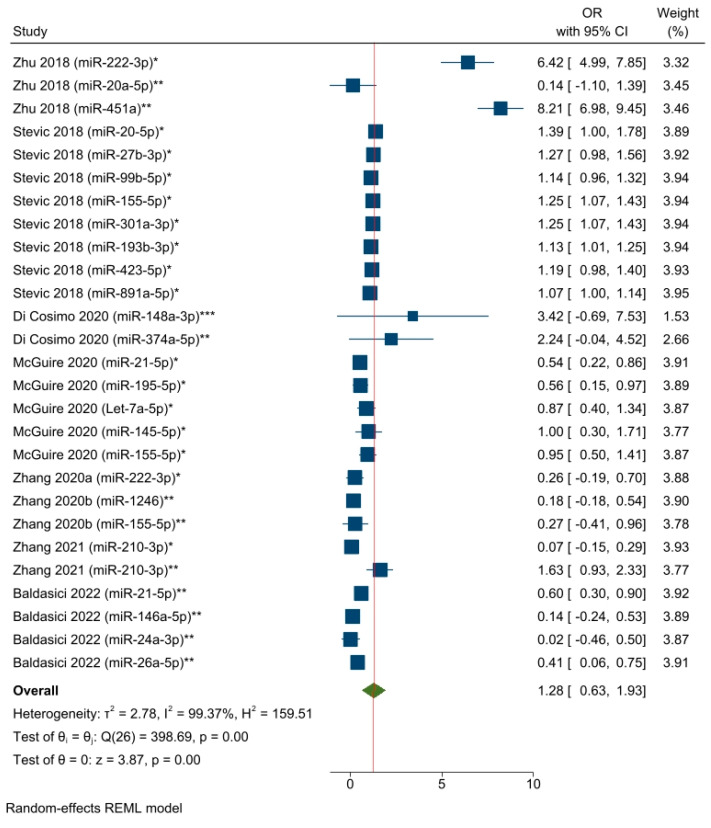
The associations between specific circulating miRNAs and the response to NAC among BC patients (corresponding to a pooled estimate based on the logistic regression models). * miRNA level before NAC; ** miRNA level during NAC; *** Changes in miRNA level from before NAC to a subsequent timepoint. Zhu 2018 [55], Stevic 2018 [56], Di Cosimo 2020 [57], McGuire 2020 [54], Zhang 2020a [58], Zhang 2020b [59], Zhang 2021 [60], Baldasici 2022 [61].

Eight studies (corresponding to 27 effect size results) analyzed the associations between specific circulating miRNAs and the response to NAC (Figure 3). All the results were obtained from univariate logistic regression models, except for two studies [55,58] that utilized multivariate logistic regression [67]. The overall pooled OR results included different circulating miRNAs detected from blood samples (i.e., serum, plasma, and whole blood) at different time points (as a single detection before starting or during the first cycles of NAC and changes in miRNA levels from multiple detections) in a mixed BC population. The selected miRNAs were found to be associated with the pCR rate (OR = 1.28, 95% CI 0.63–1.93, SE = 0.33, *p* < 0.05). The use of a random effect model was plausible due to high heterogeneity across the studies, i.e., I^2^ = 99.37%, and the Q-statistic was significant (chi^2^ (26) = 398.69; *p* < 0.05). Unfortunately, the performance of a subgroup analysis or meta-regression was not feasible given the small number of eligible studies and the limited commonality between the studies’ variables. To evaluate the impact of probable outliers on the calculation of the overall effect size, we ran a leave-one-out sensitivity meta-analysis (Supplemental Figure S2). The removal of the first and third results [55] appeared to have a more significant effect (compared to other studies) on assessing the total effect size. Specifically, omitting the first result reduced the overall ratio by around 0.2, while eliminating the third result decreased the overall ratio by nearly 0.3. In addition, we conducted a sensitivity analysis by excluding the results concerning miR-21-5p and miR-155-5p, as the predictive roles of these circulating miRNAs were separately analyzed. The aim was to assess the predictive significance of the remaining miRNAs. The analysis showed results that were similar to the general estimation: OR = 1.44, 95% CI 0.63–2.25, and *p* < 0.05 (Supplementary Figure S3).

**Figure 4:**
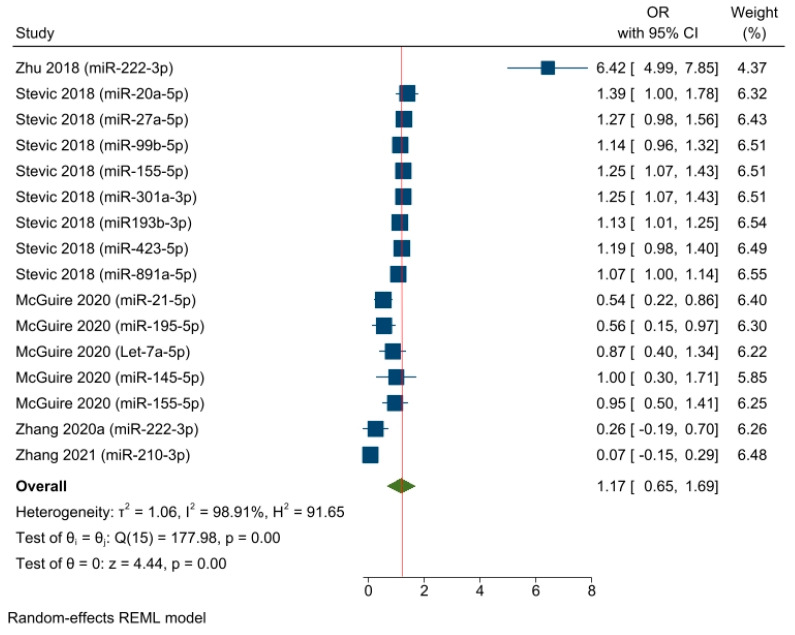
The associations between baseline miRNA detection and the response to NAC among BC patients (pooled estimate based on the logistic regression models). Zhu 2018 [55], Stevic 2018 [56], McGuire 2020 [54], Zhang 2020a [58], Zhang 2021 [60].

A separate OR meta-analysis (including five studies with a total of sixteen effect sizes) was conducted based on the timepoint of sample collection, selecting results that pertained to baseline miRNA detection (i.e., before starting NAC) (Figure 4). The OR pooled estimate of specific baseline miRNA levels demonstrated an objective response in the neoadjuvant setting among a mixed population of BC patients (OR = 1.17, 95% CI 0.65–1.69, SE = 0.26, *p* < 0.05). However, the timepoint variable did not explain the statistical heterogeneity, which remained high, as indicated by an I^2^ value of 98.91% and a significant Q-statistic (chi2 (15) = 177.98; *p* < 0.05).

### 3.4. Pooled Estimates of the AUC of the ROC Curve Models

**Figure 5:**
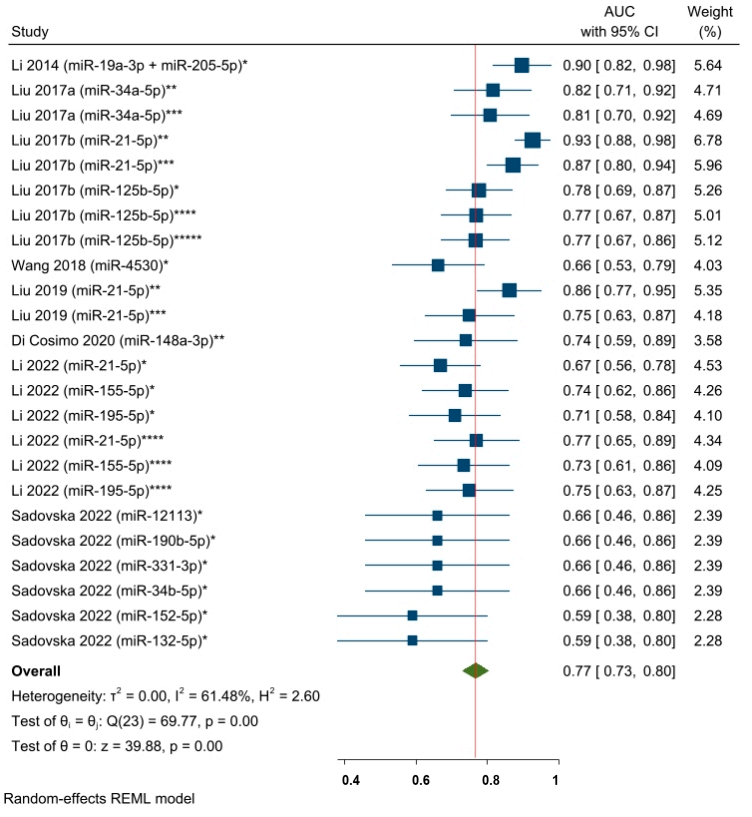
The predictive power of specific circulating miRNAs and the response to NAC among BC patients (pooled estimate based on AUCs). * miRNA level before NAC; ** Changes in miRNA levels from before NAC to a subsequent timepoint during NAC; *** Changes in miRNA levels from before NAC to a subsequent timepoint at the end of NAC; **** miRNA level during NAC; ***** miRNA level at the end of NAC. Li 2014 [62], Liu 2017a [63], Liu 2017b [28], Wang 2018 [64], Liu 2019 [26], Di Cosimo 2020 [57], Li 2022 [65], Sadovska 2022 [66].

Eight studies (corresponding to twenty-four effect size results) analyzed the predictive power of specific circulating miRNAs and the response to NAC in a mixed population of BC patients (Figure 5). The overall pooled AUC results included specific miRNAs expressed at different timepoints (as a single detection before starting or during the first cycles of NAC and changes in miRNA levels from multiple detections) in serum and plasma samples. The pooled AUC value was 0.77, 95% CI 0.73–0.80, and *p* < 0.05, which indicates an acceptable predictive miRNA performance with respect to NAC response. Moderate to substantial statistical heterogeneity was found across the studies, i.e., I^2^ = 61.48% and a significant Q-statistic was found (chi2 (23) = 69.77; *p* < 0.05). According to the leave-one-out sensitivity analysis, no study significantly influenced the overall effect size (Supplementary Figure S4). Furthermore, a sensitivity analysis was performed by excluding the circulating miR-21-5p and miR-155-5p results, as their predictive roles were individually meta-analyzed. The objective was to assess whether there would be any benefit in evaluating the remaining miRNAs. The pooled AUC value showed results that were similar to the general estimation: AUC = 0.75, 95% CI 0.71–0.79, and *p* < 0.05 (Supplementary Figure S5).

**Figure 6:**
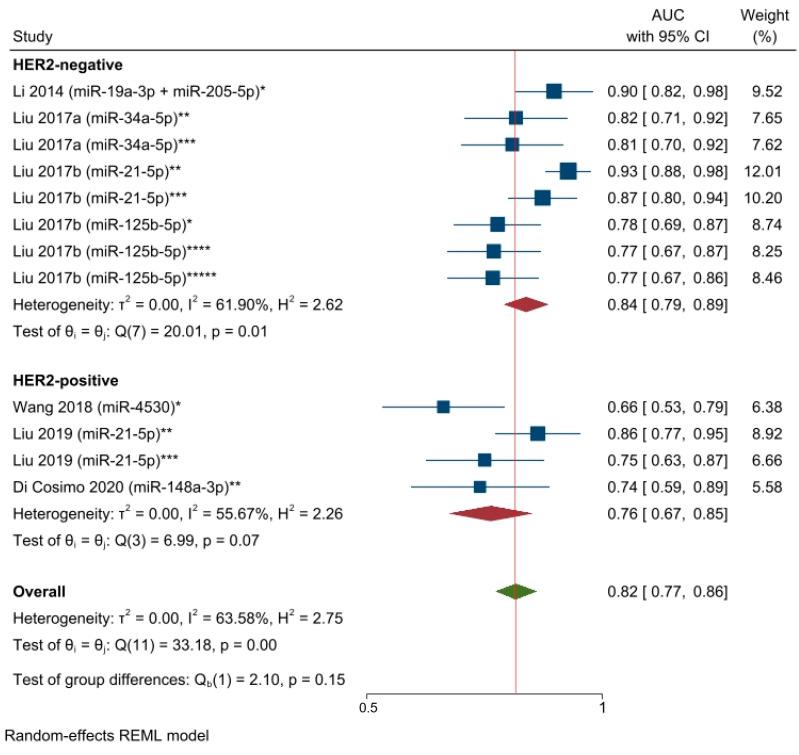
The predictive power of specific circulating miRNAs and the response to NAC among HER2-negative and HER2-positive breast cancer patients (AUCs pooled estimate). * miRNA level before NAC; ** Changes in miRNA levels from before NAC to a subsequent timepoint during NAC; *** Changes in miRNA levels from before NAC to a subsequent timepoint at the end of NAC; **** miRNA level during NAC; ***** miRNA level at the end of NAC. Li 2014 [62], Liu 2017a [63], Liu 2017b [28].

Despite the limited number of available studies, we performed a subgroup analysis based on BC molecular subtypes by excluding two studies [65,66] that included both HER2-positive and HER2-negative molecular subtypes. The selected circulating miRNAs showed higher significant statistical predictive performance for the HER2-negative patients than for the HER2-positive molecular subtype: AUC = 0.84, 95% CI 0.79–0.89, and *p* < 0.05; AUC = 0.76, 95% CI 0.67–0.85, and *p* = 0.07. However, the results of test of group differences were not significant (Q = chi (1) = 2.10; *p* = 0.15). Thus, the miRNAs’ predictive performance was not statistically significant within the two BC patients’ groups (Figure 6).

**Figure 7:**
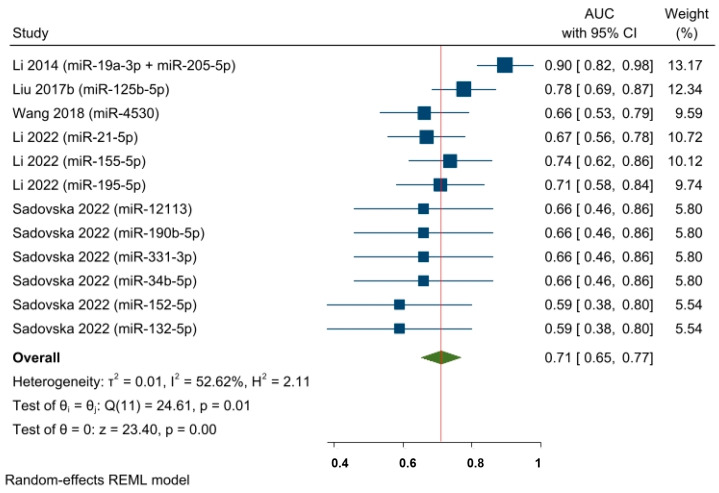
The predictive power of specific baseline miRNAs’ expression and the response to NAC among BC patients (pooled estimate based on AUCs). Li 2014 [62], Liu 2017b [28], Wang 2018 [64], Li 2022 [65], Sadovska 2022 [66].

An additional AUC meta-analysis (concerning five studies and twelve effect sizes) was conducted based on baseline miRNA detection (i.e., before NAC) (Figure 7). The AUC pooled estimate of specific baseline miRNA levels was shown to predict responses in a neoadjuvant setting among a mixed population of BC patients (AUC = 0.71, 95% CI 0.65–0.77, and *p* < 0.05). However, the statistical heterogeneity remained moderate (I^2^ = 52.62% and significant Q-statistic (chi2 (11) = 24.61; *p* < 0.05)).

**Figure 8:**
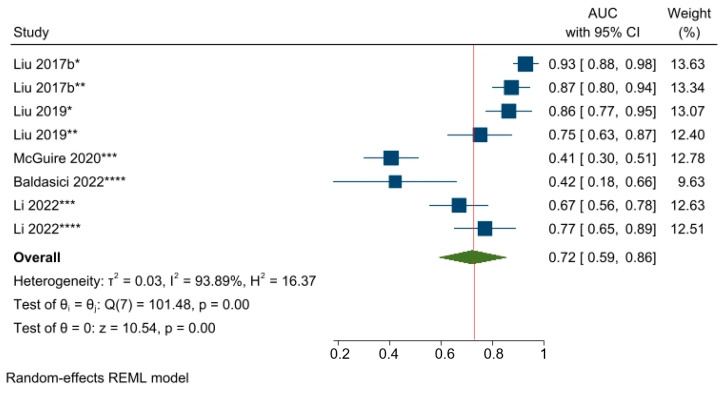
The predictive power of miR-21-5p and the response to NAC among BC patients. * Changes in miRNA levels from before NAC to a subsequent timepoint during NAC; ** Changes in miRNA levels from before NAC to a subsequent timepoint at the end of NAC; *** miRNA level before NAC; **** miRNA level during NAC. Liu 2017b [28], Liu 2019 [26], McGuire 2020 [54], Baldasici 2022 [61], Li 2022 [65].

The predictive power of circulating miR-21-5p, the most commonly studied miRNA from among the studies on predicting responses to NAC, was examined by converting the OR estimates of two studies [54,61] into AUC values. The pooled AUC value was 0.72 (95% CI 0.59–0.86, *p* < 0.05), which suggests that miR-21-5p has an acceptable ability to predict NAC responses among a mixed population of BC patients (Figure 8).

**Figure 9:**
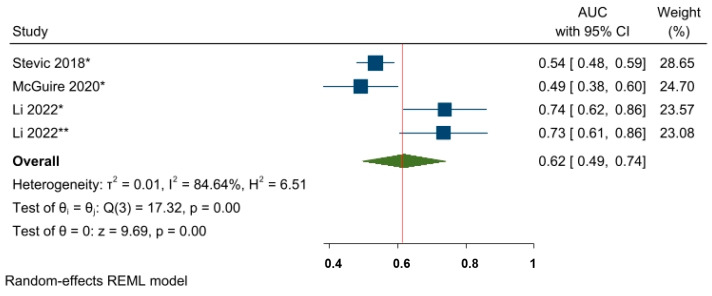
The predictive power of miR-155-5p and the response to NAC among BC patients. * miRNA level before NAC; ** miRNA level during NAC.

Furthermore, although only three eligible studies and four effect sizes were included, the prognostic value of miR-155-5p was also investigated by converting the OR estimates into AUC values. The pooled AUC value was 0.62, 95% CI 0.49–0.74, and *p* < 0.05, indicating that miR-155-5p has an almost acceptable ability to predict NAC responses among BC patients (Figure 9).

We did not assess publication bias with funnel plot analysis since our meta-analysis included a maximum of nine studies, and the inclusion of fewer than ten studies may not provide enough power to test the real asymmetry [68]. Stevic 2018 [56], McGuire 2020 [54], Li 2022 [65].

## 4. Discussion

The literature has extensively documented the potential role of circulating miRNAs as biomarkers for the diagnosis, prognosis, and treatment response prediction of BC [24,25,69,70,71]. However, a considerable disparity in the research methodology has been identified, thus preventing the adoption of circulating miRNAs as a biomarker into clinical practice. This is the first quantitative synthesis, incorporating logistic regression and ROC curve analysis, that guides researchers and clinicians regarding the predictive performance of circulating miRNAs in determining responses to NAC among BC patients.

Our summary of the analyzed OR results revealed that the majority of circulating miRNAs, which had been studied in recent clinical trials, exhibited an association between pCR among BC patients undergoing NAC. However, a strong association cannot be verified, as the OR magnitude exceeds 1.0. Specifically, the likelihood of obtaining a response improves as the level of selected circulating miRNA increases. Similar results were found with the baseline detection of the same miRNAs. The sensitivity analysis of the OR meta-analysis suggested a considerable influence of Zhu’s study [55] on the overall effect size. This effect was probably due to the key role of specific miRNAs (miR-222-3p and miR-451a) in detecting responses among HER2-negative BC patients [72,73].

The pooled AUC analysis demonstrated the overall acceptable performance of the investigated miRNAs with respect to predicting the response to NAC in the same study population. This was observed for miRNAs that were detected at baseline and at different time points. Nevertheless, these pooled results included different circulating miRNAs, diverse types of samples, and a heterogeneous BC population, which might have influenced the magnitude of the overall effect size since the consideration of molecular subtype is crucial in determining specific miRNAs’ predictive and prognostic value [74,75,76,77]. In addition, specific miRNAs’ level patterns may influence a predictive biomarker’s ability to predict a therapeutic response [27,78]. Differences in treatment regimens may also impact biomarkers’ predictive performance [79].

NAC has emerged as a pivotal treatment approach for early BC. Previous neoadjuvant trials included patients across all BC subtypes, who were primarily selected based on tumor size and nodal involvement. These trials demonstrated significant survival benefits, particularly among patients with HER2+ breast cancer and TNBC [4]. Subsequent studies focused on “targeted” therapies tailored to specific tumor subtypes, further validating the prognostic significance of pCR in HER2-positive breast cancer and TNBC. As a result, NAC with anti-HER2 targeted therapy for HER2-positive tumors and NAC with pembrolizumab for TNBC have become standard treatments for these subtypes [80,81]. However, managing HR+/HER2-negative (luminal) BC remains a challenge. For the treatment of this subtype, NAC may be proposed to achieve downsizing for breast-conserving surgery (BCS) after a comprehensive multidisciplinary evaluation, provided there are no contraindications to BCS [82]. By delving into these aspects, we can gain a deeper understanding of the clinical relevance of our findings and identify specific patient populations for whom our results may have significance. To achieve this, we conducted a subgroup analysis based on BC molecular subtypes, specifically HER2-positive and HER2-negative subtypes. Despite the higher predictive performance of the selected circulating miRNAs for HER2-negative patients compared to those with the HER2-positive subtype, no statistically significant differences between the two groups emerged. However, compelling evidence exists in the literature in this research area indicating significant associations between the expression of miR-21-5p and residual disease [83] and resistance to trastuzumab [84]. miR-21-5p influenced the response to trastuzumab-based treatment by initiating an IL-6/STAT3/NF-κB-mediated signaling loop and activating the PI3K pathway [83]. Conversely, trastuzumab upregulated miR-155-5p, demonstrating potent downregulation and suppression of ErbB2-induced malignant transformation of breast epithelial cells [85].

Despite the methodological limitations of a comprehensive quantitative analysis involving multiple miRNAs, our findings from both models support the promising role of selected circulating miRNAs, namely, miR-21-5p and miR-155-5p, in predicting responses to NAC in a mixed population of BC patients. Specifically, lower levels of circulating miR-21-5p and miR-155-5p (evaluated at baseline and as a change from a timepoint during or after NAC compared to the baseline) were observed among BC patients with pCR compared to those with residual disease in the studies included the meta-analysis [26,28,54,56,59,61,65]. Thus, a low level of these two circulating miRNAs could help to identify patients who will benefit from NAC. Specifically, baseline miRNA levels provide insights into the pre-treatment status of a patient and may serve as predictive factors for NAC response. By analyzing miRNA profiles before initiating treatment, it may be possible to identify patients that are more likely to respond favorably or those who might experience resistance to therapy. Such information can aid in individualizing treatment decisions and optimizing patient outcomes. On the other hand, evaluating miRNA levels during NAC introduces a different perspective. Changes in circulating miRNAs following treatment reflect the dynamic response of a tumor to a therapy. These alterations can indicate the effectiveness of the corresponding treatment and help to assess the clinical response during treatment. Therefore, they can serve as potential biomarkers for monitoring therapeutic response. It is crucial to consider the clinical relevance of miRNA expression levels when making decisions about ongoing NAC. While intermediate circulating miRNA levels may be associated with pCR, they may not always provide adequate information with which to guide treatment decisions if chemotherapy has already been initiated. Furthermore, understanding the biological significance of miRNA level changes during NAC is paramount. It is necessary to differentiate miRNAs directly affected by chemotherapy from those that exhibit parallel changes due to the treatment’s systemic effects. This distinction can shed light on the mechanisms of action and potential roles of specific miRNAs in chemotherapy response and help identify miRNAs that may be valuable therapeutic targets.

Considering the limited number of studies included, our findings should be interpreted cautiously. However, the present results are consistent with the existing literature. Indeed, plasma miR-21-5p, miR-155-5p, miR-10b-5p, and miR-let-7a-5p have recently been described as potential biomarkers for monitoring BC patients’ outcomes [86]. Similarly, in a recent study by Chekhun and colleagues (which was not included in the present meta-analysis due to the lack of OR/AUC determination), serum miR-21-5p levels were associated with response to neoadjuvant polychemotherapy in luminal B tumors (i.e., lower miRNA levels were associated with increased sensitivity) [87]. In particular, the predictive effects of miR-21-5p and miR-155-5p were roughly similar. However, the limited number of studies prevents a plausible interpretation of these findings.

Furthermore, since pCR has been suggested to be a surrogate endpoint for long-term outcomes [4,5], we could hypothesize that circulating miR-21-5p and miR-155-5p might also be helpful biomarkers for prognostication among BC patients undergoing NAC. Accordingly, a recent meta-analysis identified plasma miR-155-5p, miR-133a-3p, miR-21-5p, and miR-205-5p as prognostic and follow-up markers for BC patients [88].

MiR-21-5p and miR-155-5p are two of the most investigated miRNAs in relation to BC and seem to play a key role in BC development, BC progression, and BC patients’ responses to therapy (Supplementary Figure S6). In particular, miR-21-5p is a well-known oncogenic miRNA involved in BC tumor growth and metastasis formation. In vitro and in vivo studies have demonstrated that the inhibition of this miRNA impaired tumor progression by increasing apoptosis and suppressing angiogenesis through the inhibition of the HIF-1A/VEGF/VEGFR2-associated signaling pathway [89,90]. In addition, it has been shown that miR-21-5p exerts its oncogenic role by targeting multiple tumor suppressor genes, such as Bcl-2, TPM1, PDCD4, and PTEN [90,91,92,93]. Similarly, also miR-155-5p is an oncogenic miRNA capable of promoting tumor growth, angiogenesis, and BC aggressiveness [93,94]. In fact, in vitro and in vivo studies have demonstrated that miR-155-5p can increase BC growth by inhibiting SOCS1, thus upregulating STAT3 signaling [95] and promoting angiogenesis and metastasis formation through targeting VHL [96]. Other targets of miR-155-5p include tumor suppressor genes such as FOXO3a, the Ras homolog gene family member A (RhoA), and TP53NP1 [97,98,99]. Finally, as stated above, it has been found that both miRNAs play a role in chemotherapy response [83,85].

The investigation of the involvement of miRNAs in cancer development and progression and their possible use as cancer biomarkers began with their examination in cancer tissues, which shed light on their role in cancer development, progression, and therapeutic response, including with respect to BC [6,11,12,13,14]. For this purpose, many studies have taken advantage of the Cancer Genome Atlas (TCGA) database [100,101,102,103,104] and consistently shown a higher expression of miR-21-5p and miR-155-5p in BC tissues compared to normal tissues (*p* = 0.001 and *p* < 0.0001) [100,103,104]. However, the prognostic roles of both tissue miRNAs have yielded conflicting results, even within the TCGA-based investigations [100,101,102,103,104]. Specifically, high expression levels of tissue miR-21-5p and miR-155-5p were found to be associated with poor prognosis among BC patients in some studies (Hazard Ratio [HR] = 1.63 [95% CI: 1.17–2.28], *p* = 0.0038; HR = 1.33 [95% CI: 1.09–1.63], *p* = 0.0047, respectively) [100,104], whereas in other investigations, high levels of both tissue miRNAs were found to be significantly associated with longer overall survival (*p* = 0.0048 and 0.048, respectively for the two miRNAs) [101,103]. Interestingly, Pasculli and colleagues found no statistically significant associations between miR-155-5p expression and BC patients’ prognosis in the TCGA database, although they found that miRNA levels were associated with unfavorable prognostic factors, such as high tumor stages, reduced expression of ER and PgR, and high Ki-67 expression [102]. The inconsistencies among these studies may be attributed to the different cut-off values used for dichotomizing miRNA levels. Therefore, further studies incorporating more standardized methodological approaches are warranted to explore the prognostic role of tissue miR-21-5p and miR-155-5p in BC, particularly using TCGA data. Besides TCGA-based studies, different authors have analyzed the association of tissue miR-21-5p and miR-155-5p with BC prognosis [105,106,107]. A recent meta-analysis conducted by Bahramy and colleagues demonstrated a significant association between poor overall survival and miR-21-5p expression in BC tissues (HR = 1.93 [95% CI: 1.62–2.30] *p* = 0.02) [105]. Similarly, in the updated meta-analysis conducted by Wu et al., miRNA-155-5p was found to be significantly associated with poor overall survival among cancer patients (HR = 1.38 [95% CI: 1.25–1.54] *p* < 0.001), although this association was not maintained in a BC subgroup analysis [106]. Notably, another recent meta-analysis focusing on TNBC subtype revealed that high miR-21-5p expression and low miR-155-5p tissue levels were associated with worse prognosis in terms of overall survival (HR: 2.56 [95% CI: 1.49–4.40], *p* = 0.0007; HR: 0.68 [95% CI: 0.58–0.81], *p* < 0.00001) [107]. Thus, understanding the intricate relationship between circulating and tissue miRNAs in BC, and their potential implications for prognosis, requires comprehensive exploration.

The present meta-analysis has several limitations. First, we performed an overall meta-analysis by combining different circulating mRNAs to quantify the association between miRNAs and responses to NAC, thereby preventing the accurate identification of the specific miRNA that best predicts treatment responses in the selected population. However, this meta-analysis aims to offer a comprehensive quantitative overview of the existing evidence in this field rather than a precise directive for healthcare professionals in daily clinical practice. Furthermore, we attempted to pool the evidence to analyze at least the most relevant miRNAs in the studies. Second, the present study’s methodology provides a descriptive synthesis of published associations between specific circulating miRNAs and response to NAC. This approach did not involve amalgamating the raw data from the primary studies. The high variability in terms of the characteristics of the patients, their disease stages, their molecular cancer subtypes, and the type of blood sample used could call into question the plausibility of the results. The impact of pre-analytical and analytical variables, as well as patient-related factors, on the analysis of circulating miRNA has been the subject of extensive debate. These factors have been known to generate artefacts that can significantly affect such analyses [21,25,108,109,110,111,112]. Specifically, we could detect distinct circulating miRNAs and different miRNA levels in different biological fluids, and whole blood may be strongly contaminated by miRNAs contained in blood cells [113,114,115,116,117]. In addition, individual characteristics such as age, ethnicity, concomitant medications, smoking habits, diet, and physical activity have been shown to influence circulating miRNA levels [108,118,119,120,121,122,123,124]. Third, there was a great deal of heterogeneity in the outcome definitions. In certain investigations, pCR was used as a binary variable denoting the presence or absence of residual invasive disease after NAC. In contrast, in other investigations, responses were assessed based on the definition of a complete response, a partial response, stable disease, and progressive disease. All these variables may at least partly explain the heterogeneity in our meta-analysis. Fourth, despite the scarcity of studies, we analyzed the miRNAs’ performance within two groups of BC patients (HER2-positive and HER2-negative). As a result, the statistical power of the effect computations was reduced. Fifth, meta-analyses of prediction models are recommended if more than five external validation studies for the same index prognostic model are available [40]. Nevertheless, as data from multiple studies were unavailable for external validation, we only included a few validated studies to assess the ability of miR-21-5p and miR-155-5p, resulting in lower statistical power for the overall effect estimation. In this regard, incorporating validation cohorts in the selected primary studies could contribute to achieving more consistent results [125]. However, this systematic review including a meta-analysis aims to give a quantitative overview of the association between specific circulating miRNAs and the response to neoadjuvant therapy rather than corroborate a validated model for specific miRNAs’ predictive ability. Sixth, we included univariate analysis results in our analysis as only two studies reported multivariate analysis results. The real value of circulating miRNAs is determined by their contribution to clinical and pathological factors, such as tumor stage or molecular subtype, that predict pCR among BC patients treated with NAC. Then, a large part of the observed effect for circulating miRNAs may be associated with some of these independent variables. However, multivariate meta-analyses are recommended in the case of post-estimation modelling that requires effect estimates for multiple correlated outcomes [66,126]. Although multivariate models generally provide more precise estimates, the point estimates from both multivariate and univariate models are often comparable [66,127]. Seventh, the expression of the data outcomes varied across the included studies: two studies [54,61] provided associations as OR per unit increase in the miRNA level rather than expressing dichotomized data outcomes (i.e., high versus low miRNA levels). In addition, the results for dichotomized high versus low miRNA levels used different cut-offs. Although the outcome measure was the same across the pooled primary studies (i.e., OR), combining dichotomous and continuous data outcomes and utilizing different cut-offs may result in conflicting and potentially biased results. However, the variability in these effects should be taken into account, and the OR meta-analysis results indicate the strength of the associations between the biomarkers and the response to NAC. Lastly, it is important to note that the current evidence based on high-quality research is limited, as 40% of the studies have a combination of high and unclear bias risks.

## 5. Conclusions

To the best of our knowledge, this is the first systematic review and meta-analysis that provides a comprehensive, quantitative synthesis of the predictive value of circulating miRNAs with respect to NAC response among BC patients. Overall, our findings support the hypothesis that miRNAs may play a significant role in predicting the response to NAC among BC patients. Specifically, we found that circulating miR-21-5p and miR-155-5p may serve as predictive biomarkers in the neoadjuvant setting among BC patients. However, due to the small number of studies included in the meta-analysis and the high degrees of clinical and statistical heterogeneity observed, further clinical studies are required in order to assess the predictive power of circulating miR-21-5p and miR-155-5p. In addition, further high-quality research is needed to selectively assess other circulating miRNAs’ capacity to serve as potential biomarkers for BC in the neoadjuvant setting.

## Figures and Tables

**Table 1:** Summary of the main characteristics of the studies included in meta-analyses. * We reported “standard of care” when the paper’s authors did not provide any detail on the chemotherapy regimen.

ID in Meta-Analyses	BC Subtype	BC Patient n.	BC Patient Gender	Sample	Quantification Method	Outcome Data Expression	Blood Collection Timing	NAC	Study Location	Reference Number
Zhu 2018	All	109	Female	Plasma	qRT-PCR	High versus low miRNA levels	At baseline, after two cycles of NAC, and before surgery	Epirubicin + Paclitaxel 1,2q21	China	[55]
Stevic 2018	HER2+ and TNBC	435	Female	Plasma (exosome)	qRT-PCR	High versus low miRNA levels	At baseline (also before surgery for 9 patients)	Weekly Paclitaxel + non-pegylated liposomal doxorubicin +/− Carboplatin, plus Trastuzumab, for HER2+ patients	Germany	[56]
Di Cosimo 2020	HER2+	52	Female	Plasma	qRT-PCR	High versus low miRNA levels	At baseline, after two weeks of treatment, prior to surgery, and eventually at the time of relapse	Lapatinib + weekly trastuzumab, or lapatinib + weekly trastuzumab + weekly paclitaxel	122 study locations (worldwide)	[57]
McGuire 2020	All	114	Female	Whole blood	qRT-PCR	OR per unit increase in the miRNA level	At baseline	Standard of care *	Ireland	[54]
Zhang 2020a	HER2+	65	Female	Serum	qRT-PCR	High versus low miRNA levels	At baseline	Weekly Paclitaxel + cisplatin, plus Trastuzumab for HER2+ patients	China	[58]
Zhang 2020b	HER2+	107 early-stage (+68 metastatic)	Female	Plasma (exosome)	qRT-PCR	High versus low miRNA levels	During/after NAC	Triweekly or weekly trastuzumab	China	[59]
Zhang 2021	Luminal B	37	Female	Serum	qRT-PCR	High versus low miRNA levels	At baseline and after two/four cycles of NAC	Taxane- and/or anthracycline-based regimen, plus Trastuzumab for HER2+ patients	China	[60]
Baldasici 2022	HR+	72	Female	Plasma (extracellular vesicles)	q-PCR	OR per unit increase in miRNA level	At baseline	Standard of care *	Romania	[61]
Li 2014	HR+	68	Female	Serum	RT-PCR	High versus low miRNA levels	At baseline	Epirubicin + Paclitaxel 1,2q21	China	[62]
Liu 2017a	HER2-	86	Female	Serum	qRT-PCR	High versus low miRNA levels	At baseline, at the end of the second cycle, and at the end of NAC	Docetaxel+ Epirubicin + Cyclophosphamine 1q21	China	[63]
Liu 2017b	HER2-	118	Female	Serum	qRT-PCR	High versus low miRNA levels	At baseline, at the end of the second cycle, and at the end of NAC	Docetaxel+ Epirubicin + Cyclophosphamine 1q21	China	[28]
Wang 2018	All	78	Not specified	Serum	qRT-PCR	High versus low miRNA levels	At baseline	Taxane- and anthracycline-based NAC	China	[64]
Liu 2019	HER2+	83	Female	Serum	qRT-PCR	High versus low miRNA levels	At baseline, at the end of the second cycle, and at the end of NAC	Docetaxel + Paraplatin +Trastuzumab 1q21	China	[26]
Li 2022	All	65	Female	Plasma	Graphene Oxide-Based qRT-PCR	High versus low miRNA levels	At baseline, at the end of each cycle of NAC, and at the end of NAC	Standard of care *	China	[65]
Sadovska 2022	All	32	Female	Plasma (extracellular vesicles)	RNA sequencing	High versus low miRNA levels	At baseline, at the end of NAC, 7 days after the surgery, and 6, 12, and 18 months after the surgery	NAC regimens containing Doxorubicin, Docetaxel, Cyclophosphamide, Paclitaxel, 5FU, and Epirubicin	Latvia	[66]

Abbreviations: BC—breast cancer; TNBC—triple-negative breast cancer; HER2—human epidermal growth factor 2; HR—hormone receptor; qRT-PCR—quantitative real-time polymerase chain reaction.

## Supplementary Materials

The following supporting information can be downloaded at: https://www.mdpi.com/article/10.3390/cancers15133424/s1, Figure S1: CHARMS 2014 Relevant items for extraction from individual studies in a systematic review of prediction models; Figure S2: Sensitivity analysis of pooled estimate derived from regression models; * miRNA level before NAC; ** miRNA level during NAC; *** changes in miRNA levels from before NAC to subsequent timepoint.; Figure S3: Sensitivity analysis of pooled estimate derived from regression models with the exclusion of the results of miR-21-5p and miR-155-5p; * miRNA level before NAC; ** miRNA level during NAC; *** changes in miRNA levels from before NAC to subsequent timepoint; Figure S4: Sensitivity analysis of the AUC of the ROC curve models; * miRNA level before NAC; ** changes in miRNA levels from before NAC to a subsequent timepoint during NAC; *** changes in miRNA levels from before NAC to a subsequent timepoint at the end of NAC; **** miRNA levels during NAC; ***** miRNA levels at the end of NAC; Figure S5: Sensitivity analysis of the AUC of the ROC curve models with the exclusion of the results regarding miR-21-5p and miR-155-5p; * miRNA levels before NAC; ** changes in miRNA levels from before NAC to a subsequent timepoint during NAC; *** changes in miRNA levels from before NAC to a subsequent timepoint at the end of NAC; **** miRNA levels during NAC; ***** miRNA levels at the end of NAC; Figure S6: Biogenesis and functions of miR-21-5p and miR-155-5p in BC; Table S1: Risk of bias and applicability assessment.
