# Supplementary material for: Unlocking the Potential of Circulating miRNAs in the Breast Cancer Neoadjuvant Setting: A Systematic Review and Meta-Analysis
Source: Cancers (Basel). 2023 Jun 30;15(13):3424. doi: 10.3390/cancers15133424 (PMC10340268; doi:10.3390/cancers15133424)
Supplement: Supplementary file 1 [file cancers-15-03424-s001.zip › Figure S1.pdf]

CHARMS 2014 Relevant items to extract from individual studies in a systematic review of prediction models

Domain	Key items	Reported on page #
**SOURCE OF DATA**	Source of data (e.g., cohort, case-control, randomized trial participants, or registry data)	
**PARTICIPANTS**	Participant eligibility and recruitment method (e.g., consecutive participants, location, number of centers, setting, inclusion and exclusion criteria)	
	Participant description	
	Details of treatments received, if relevant	
	Study dates	
**OUTCOME(S) TO BE PREDICTED**	Definition and method for measurement of outcome	
	Was the same outcome definition (and method for measurement) used in all patients?	
	Type of outcome (e.g., single or combined endpoints)	
	Was the outcome assessed without knowledge of the candidate predictors (i.e., blinded)?	
	Were candidate predictors part of the outcome (e.g., in panel or consensus diagnosis)?	
	Time of outcome occurrence or summary of duration of follow-up	
**CANDIDATE PREDICTORS (OR INDEX TESTS)**	Number and type of predictors (e.g., demographics, patient history, physical examination, additional testing, disease characteristics)	
	Definition and method for measurement of candidate predictors	
	Timing of predictor measurement (e.g., at patient presentation, at diagnosis, at treatment initiation)	
	Were predictors assessed blinded for outcome, and for each other (if relevant)?	
	Handling of predictors in the modelling (e.g., continuous, linear, non-linear transformations or categorised)	
**SAMPLE SIZE**	Number of participants and number of outcomes/events	
	Number of outcomes/events in relation to the number of candidate predictors (Events Per Variable)	
**MISSING DATA**	Number of participants with any missing value (include predictors and outcomes)	
	Number of participants with missing data for each predictor	
	Handling of missing data (e.g., complete-case analysis, imputation, or other methods)	
**MODEL DEVELOPMENT**	Modelling method (e.g., logistic, survival, neural network, or machine learning techniques)	
	Modelling assumptions satisfied	
	Method for selection of predictors **for inclusion** in multivariable modelling (e.g., all candidate predictors, pre-selection based on unadjusted association with the outcome)	
	Method for selection of predictors **during multivariable modelling** (e.g., full model approach, backward or forward selection) and criteria used (e.g., p-value, Akaike Information Criterion)	
	Shrinkage of predictor weights or regression coefficients (e.g., no shrinkage, uniform shrinkage, penalized estimation)	
**MODEL PERFORMANCE**	Calibration (calibration plot, calibration slope, Hosmer-Lemeshow test) and Discrimination (C-statistic, D-statistic, log-rank) measures with confidence intervals	
	Classification measures (e.g., sensitivity, specificity, predictive values, net reclassification improvement) and whether a-priori cut points were used	
**MODEL EVALUATION**	Method used for testing model performance: development dataset only (random split of data, resampling methods e.g. bootstrap or cross-validation, none) or separate external validation (e.g. temporal, geographical, different setting, different investigators)	
	In case of poor validation, whether model was adjusted or updated (e.g., intercept recalibrated, predictor effects adjusted, or new predictors added)	
**RESULTS**	Final and other multivariable models (e.g., basic, extended, simplified) presented, including predictor weights or regression coefficients, intercept, baseline survival, model performance measures (with standard errors or confidence intervals)	
	Any alternative presentation of the final prediction models, e.g., sum score, nomogram, score chart, predictions for specific risk subgroups with performance	
	Comparison of the distribution of predictors (including missing data) for development and validation datasets	
**INTERPRETATION AND DISCUSSION**	Interpretation of presented models (confirmatory, i.e., model useful for practice versus exploratory, i.e., more research needed)	
	Comparison with other studies, discussion of generalizability, strengths and limitations.	
