# Supplementary material for: Unlocking the Potential of Circulating miRNAs in the Breast Cancer Neoadjuvant Setting: A Systematic Review and Meta-Analysis
Source: Cancers (Basel). 2023 Jun 30;15(13):3424. doi: 10.3390/cancers15133424 (PMC10340268; doi:10.3390/cancers15133424)
Supplement: Supplementary file 1 [file cancers-15-03424-s001.zip › Table S1.pdf]

Supplementary Table1: Risk of bias and applicability assessment

Author, Year	Risk of Bias				Applicability			Overall	
	1. Participants	2. Predictors	3. Outcome	4. Analysis	1. Participants	2. Predictors	3. Outcome	**Risk of Bias**	**Applicability**
Li , 2014	+	+	+	+	+	+	+	+	+
Liu(a), 2017	+	+	+	+	+	+	+	+	+
Liu(b), 2017	+	+	+	+	+	+	+	+	+
Stevic, 2018	+	+	+	+	+	+	+	+	+
Wang, 2018	+	+	+	-	+	+	+	-	+
Zhu, 2018	+	+	+	+	+	+	+	+	+
Liu, 2019	+	+	+	+	+	+	+	+	+
Di Cosimo , 2020	+	+	+	?	+	+	+	?	+
McGuire, 2020	+	+	+	-	+	+	+	-	+
Zhang(a), 2020	+	+	+	+	+	+	+	+	+
Zhang(b), 2020	+	+	+	+	+	+	+	+	+
Zhang, 2021	+	+	+	+	+	+	+	+	+
Baldasici, 2022	?	?	?	?	?	?	?	?	?
Li, 2022	?	?	?	?	?	?	?	?	?
Sadovska, 2022	+	+	+	?	+	+	+	?	+
