# Supplementary figures and images for: Unlocking the Potential of Circulating miRNAs in the Breast Cancer Neoadjuvant Setting: A Systematic Review and Meta-Analysis
Source: Cancers (Basel). 2023 Jun 30;15(13):3424. doi: 10.3390/cancers15133424 (PMC10340268; doi:10.3390/cancers15133424)

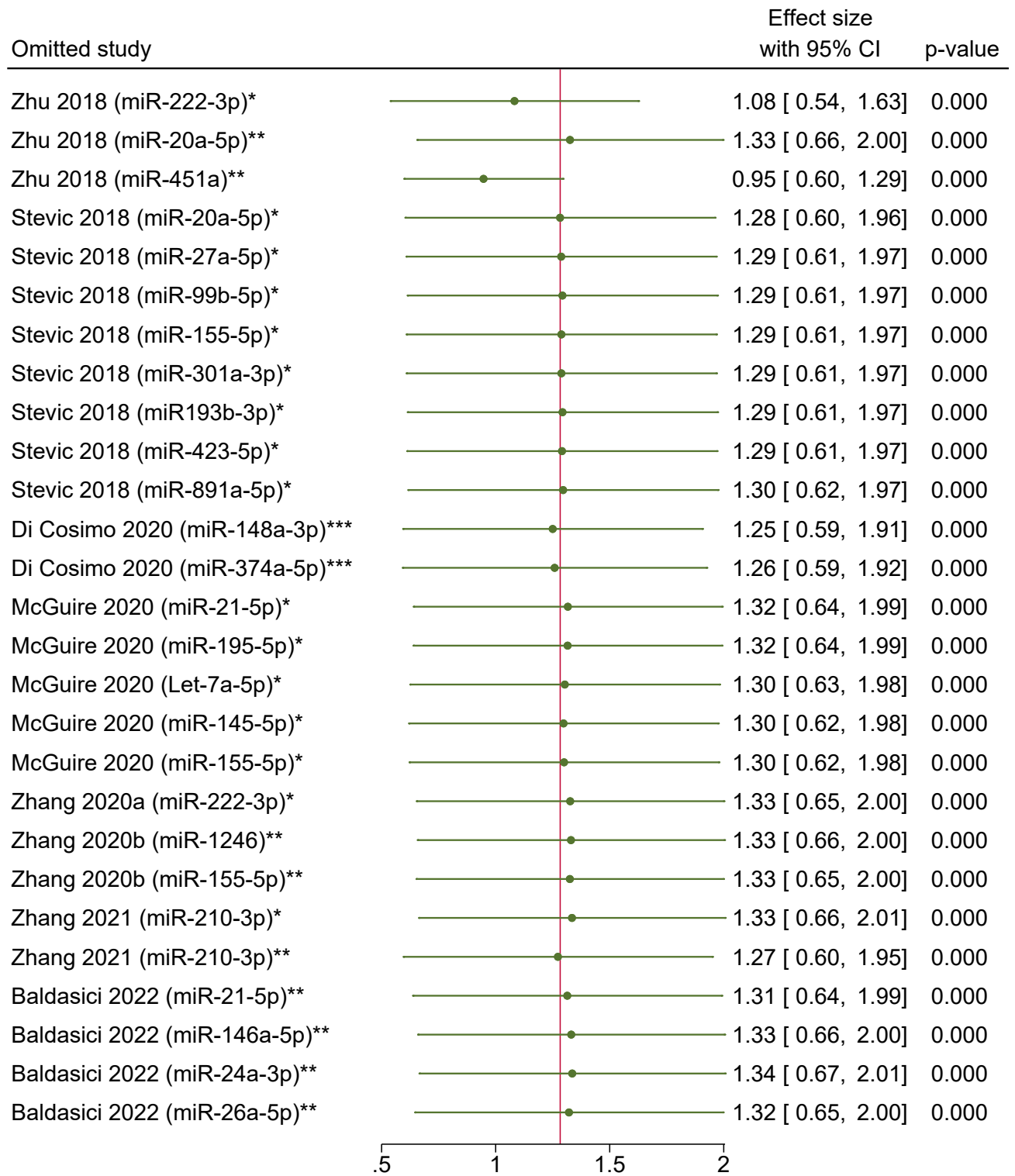

Random-effects REML model

Supplement: Supplementary file 1 [file cancers-15-03424-s001.zip › Figure S2.pdf]

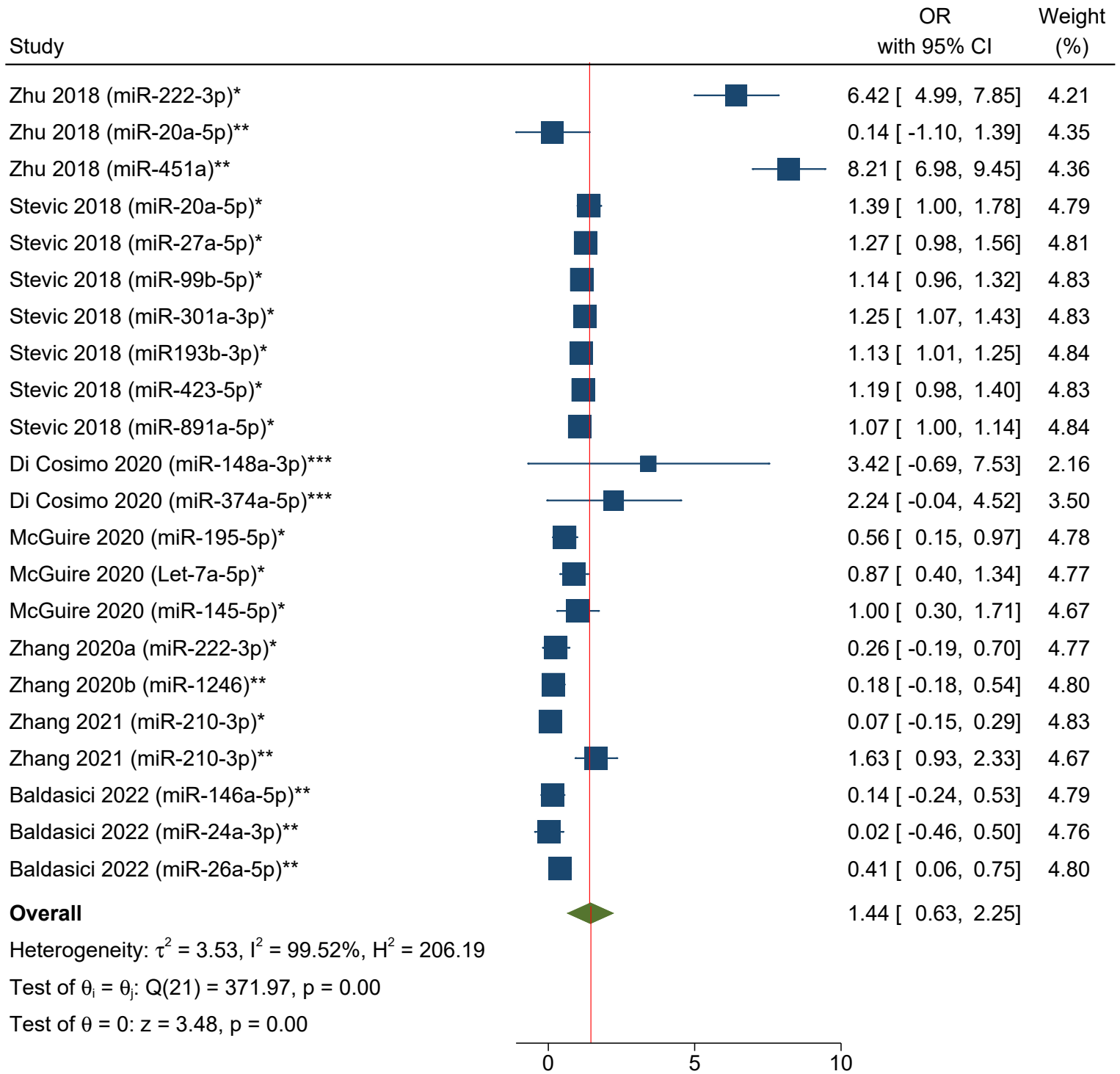

Random-effects REML model

Supplement: Supplementary file 1 [file cancers-15-03424-s001.zip › Figure S3.pdf]

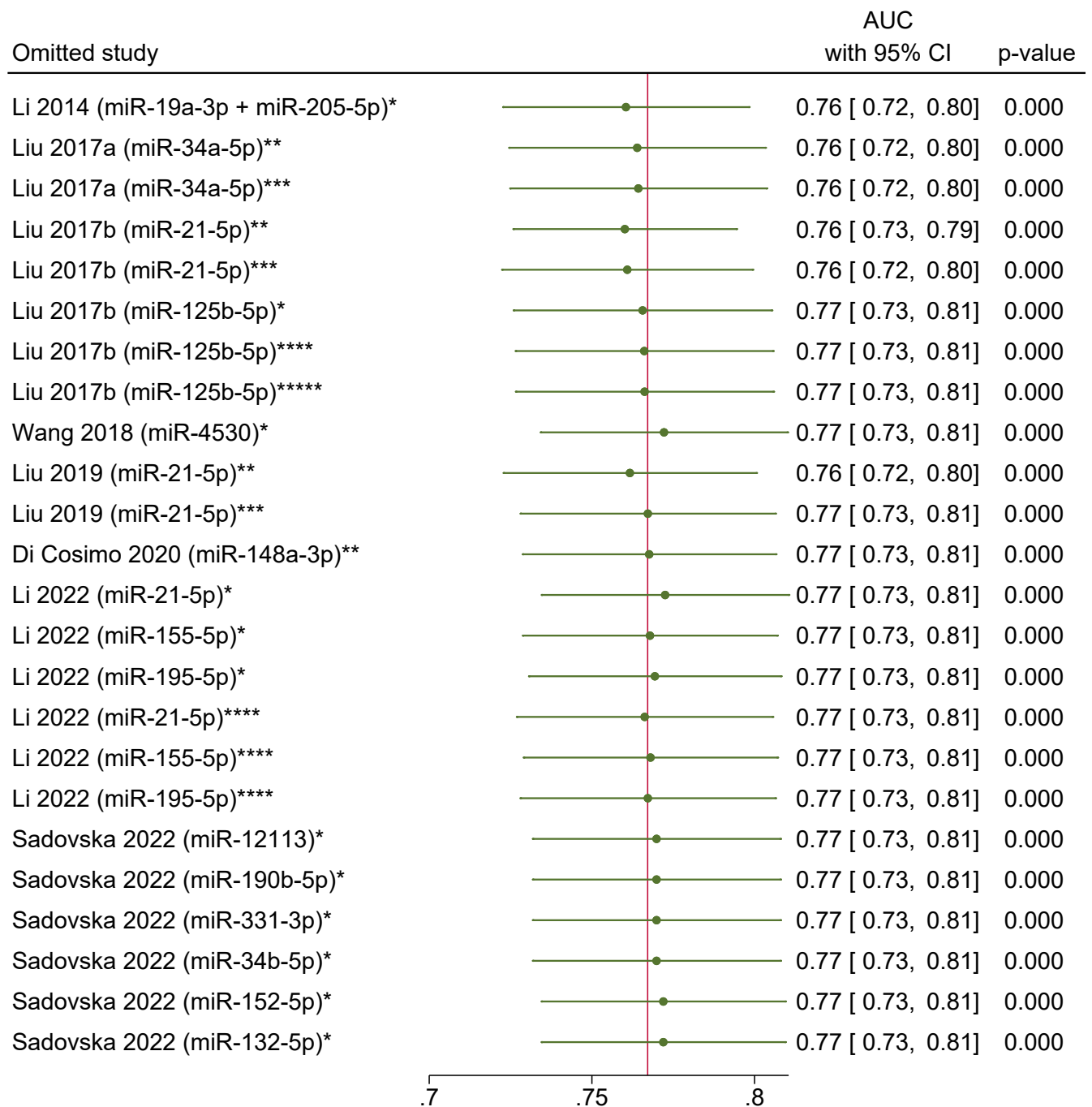

Random-effects REML model

Supplement: Supplementary file 1 [file cancers-15-03424-s001.zip › Figure S4.pdf]

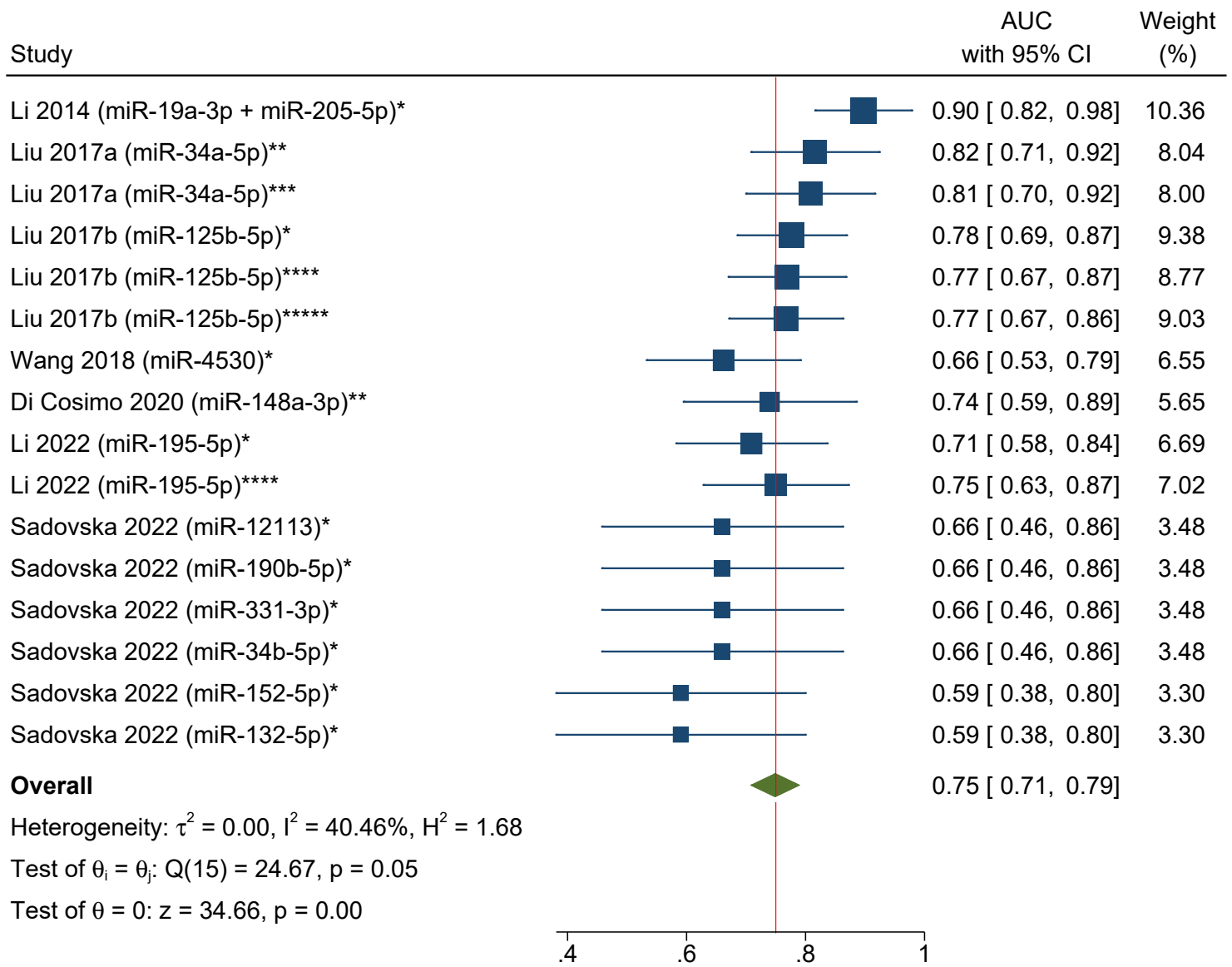

Supplement: Supplementary file 1 [file cancers-15-03424-s001.zip › Figure S5.pdf]

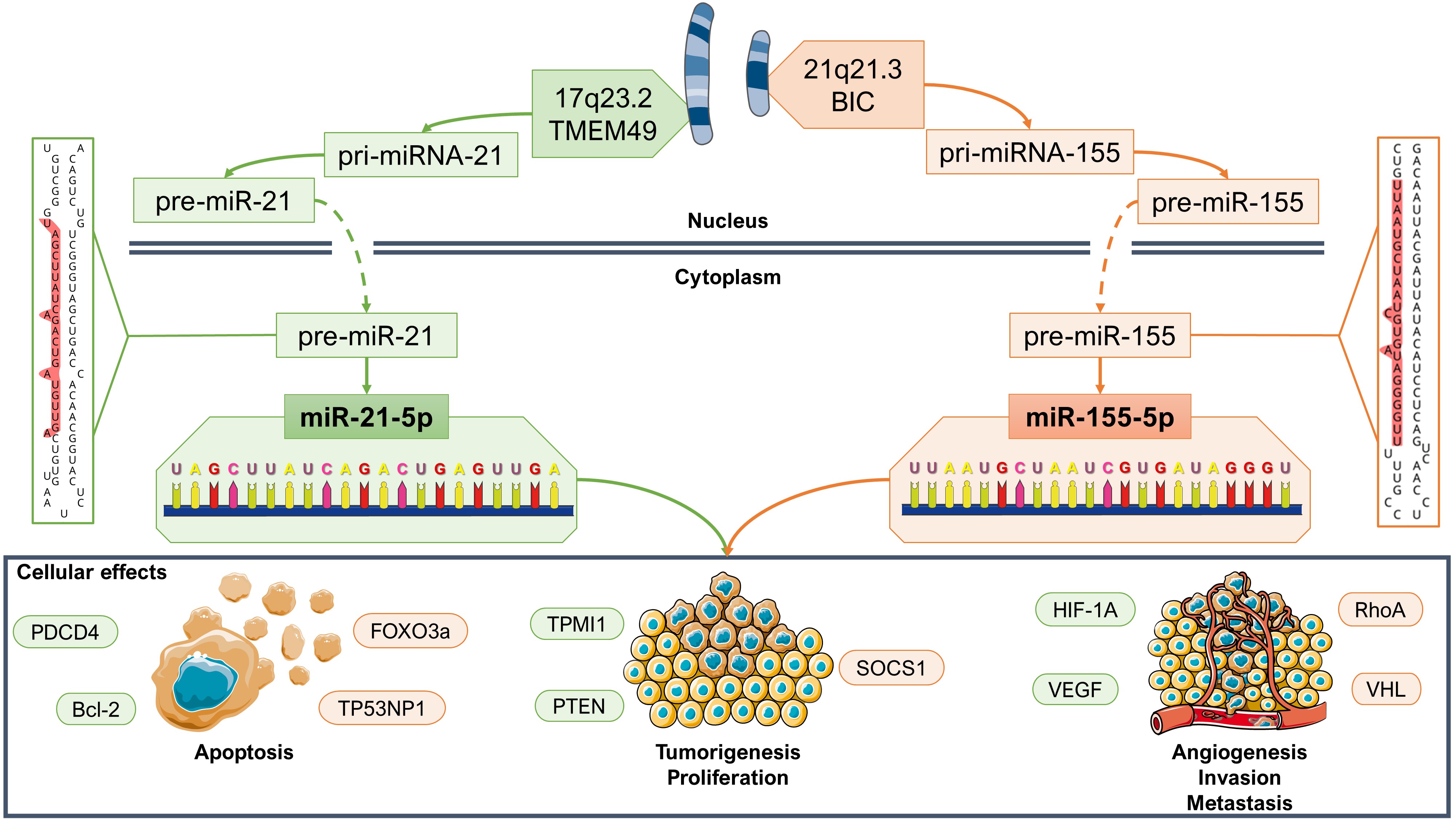

Supplement: Supplementary file 1 [file cancers-15-03424-s001.zip › Figure S6.jpg]
